# Supplementary material for: Antioxidant, Wound Healing Potential and In Silico Assessment of Naringin, Eicosane and Octacosane
Source: Molecules. 2023 Jan 20;28(3):1043. doi: 10.3390/molecules28031043 (PMC9919607; doi:10.3390/molecules28031043)
Supplement: Supplementary file 1 [file molecules-28-01043-s001.zip › molecules-2125037-supplementary.pdf]

SUPPLEMENTARY DATA

Antioxidant, Wound Healing Potential and In Silico Assessment of Naringin, Eicosane and Octacosane

Abbirami Balachandran [1], Sy Bing Choi [1], Morak-Młodawska Beata [2], Jeleń Małgorzata [2,*], Gabriele R. A. Froemming [3], Charlie A. Lavilla, Jr. [4], Merell P. Billacura [5], Stepfanie N. Siyumbwa [6] and Patrick N. Okechukwu [1,*]

[1] Department of Biotechnology, Faculty of Applied Sciences, UCSI University, Cheras, 56000 Kuala Lumpur, Malaysia
[2] Faculty of Pharmaceutical Sciences, Department of Organic Chemistry, Medical University of Sílesia, Jagiellonska, Str. 4, 41-200 Sosnowiec, Poland
[3] Basic Medical Sciences, Faculty of Medicine and Health Sciences, Universiti Malaysia Sarawak (UNIMAS), Kota Samarahan 94300, Sarawak, Malaysia
[4] Chemistry Department, College of Science & Mathematics, Mindanao State University–Iligan Institute of Technology, Iligan City, 9200, Lanao del Norte, Philippines
[5] Department of Chemistry, College of Natural Sciences and Mathematics, Mindanao State University–Main Campus, Marawi City, 9700, Lanao del Sur, Philippines
[6] Department of Pathology and Microbiology, School of Medicine, P.O. Box 50110, Lusaka, Zambia
* Correspondence: manowak@sum.edu.pl (J.M.); patrickn@ucsiuniversity.edu.my (P.N.O.); Tel.: +48-32-364-16-04(J.M.); +603-9101-8880 (P.N.O.)

Table S1 HPTLC calibration parameters

Parameters	Results (Mean ± SD) (*n* = 3)
Regression equation	y = 2.037x + 725.8
Correlation coefficient	0.973 ± 0.024
Linearity range	200 – 1000 μg/mL
Limit of detection	0.75 ± 0.29 μg
Limit of quantification	7.73 ± 0.26 μg
Amount of Naringin	642.39 ± 22.9 mg/g (64%)

Table S2 Intra- and inter-day precision of Naringin (*n* = 3)

Amount (μg/mL)	Intraday precision		Interday precision	
	SD in height	%CV	SD in height	%CV
200	13.23	0.81	23.48	1.85
600	42.58	1.11	26.56	1.08
1000	61.29	1.03	64.29	1.56

Table S3 Recovery studies of Naringin by the proposed TLC densitometric method

Concentration (μg/mL)		Sample height		Total height (sample + standard)	% Recovery
Sample	Spiked amount	Sample height	Spiked height		

600	200	2855.5	1730.7	4445.4	91.88
600	600	3162.7	3945.2	6618.6	95.40
600	1000	4979.3	86726.7	8833.1	90.40
Average					92.56

Table S4 Compounds identified in the SNP A of *M. pumilum* extract with GC-MS

No.	RT	Compounds	CAS registry	Molecular Compound	MW	PA%
1	3.762	1-Butene, 2,3,3-trimethyl-<br>2-Pentene, 4,4-dimethyl-, (Z)-<br>2-Pentene, 4,4-dimethyl-, (E)-	594-56-9 (NIST)<br>762-63-0 (NIST)<br>690-08-4 (NIST)	C_7H_{14}<br>C_7H_{14}<br>C_7H_{14}	98.186<br>98.186<br>98.186	2.16
2	14.302	2-Pentadecanone, 6,10,14-trimethyl	502-69-2 (NIST)	$C_{18}H_{36}O$	268.478	4.46
3	15.097	1-Octacosanol<br>Docos-1-ene<br>9-Nonadecene	29739970 (CS)<br>1599-67-3 (NIST)<br>31035-07-1 (NIST)	$C_{30}H_{57}F_3O_2$<br>$C_{22}H_{44}$<br>$C_{19}H_{38}$	506.768<br>308.585<br>266.505	2.61
4	15.372	Isophytol	505-32-8 (NIST)	$C_{20}H_{40}O$	296.351	2.96
5	16.877	Tetracosyl heptafluorobutyrate	29739954 (CS)	$C_{28}H_{49}F_7O_2$	550.676	2.38
6	17.661	Ethanol, 2-(octadecyloxy)-<br>1-(4-Bromobutyl)-2-piperidinone<br>1-Decanol, 2-hexyl-	2136-72-3 (NIST)<br>467195 (CS)<br>2425-77-6 (NIST)	$C_{20}H_{42}O_2$<br>$C_9H_{16}BrNO$<br>$C_{16}H_{34}O$	314.546<br>234.133<br>242.441	2.03
7	18.530	Tricosane	638-67-5 (NIST)	$C_{23}H_{48}$	324.627	2.56
9	20.659	Bis(2-ethylhexyl) phthalate<br>Bis(2-propylpentyl) phthalate	117-81-7 (NIST)<br>166657 (CS)	$C_{24}H_{38}O_4$<br>$C_{24}H_{38}O_4$	390.556<br>390.556	17.80
10	22.513	i-Propyl 9-octadecenoate<br>Octadec-9-enoic acid	111-59-1 (PC)<br>2027-47-6 (NIST)	$C_{21}H_{40}O_2$<br>$C_{18}H_{34}O_2$	324.541<br>282.461	2.46
11	23.142	Pentadecane<br>Heptadecane, 9-octyl-<br>Heptacosane, 1-chloro-	629-62-9 (NIST)<br>7225-64-1 (NIST)<br>62016-79-9 (NIST)	$C_{15}H_{32}$<br>$C_{25}H_{52}$<br>$C_{27}H_{55}Cl$	212.415<br>352.680<br>415.179	7.89
12	24.756	Heneicosane<br>Hexadecane, 1-iodo-<br>**Octacosane**	629-94-7 (NIST)<br>544-77-4 (NIST)<br>**630-02-4(NIST)**	$C_{21}H_{44}$<br>$C_{16}H_{33}I$<br>$\mathbf{C_{28}H_{58}}$	296.574<br>352.338<br>**394.760**	**11.72**
13	26.902	**Eicosane**	**112-95-8 (NIST)**	$\mathbf{C_{20}H_{42}}$	**282.548**	**5.25**
14	27.932	1,3-Dithiolane, 2-(28-norurs-12-en -17-yl)-<br>3,7,11-Trimethyl-dodeca-2,4,6,10-t etraenal<br>(22E)-3',7β-Dihydrocycloprop[7,8]-5α-ergost-22-en-3-one	473220 (CS)<br>23255776 (CS)<br>53755-18-3 (CB)	$C_{32}H_{52}S_2$<br>$C_{15}H_{22}O$<br>NR	500.885<br>218.335<br>NR	2.65
15	28.361	β –Amyrin<br>Urs-12-en-3-one	559-70-6 (NIST)<br>532729 (CS)	$C_{30}H_{50}O$<br>$C_{30}H_{48}O$	426.717<br>424.702	6.84

Table S5 List of MMPs involved in the wound healing process

Protein Name	PDB ID	Resolution

Collagenase 1 (MMP-1)	966C	1.90 Å
Collagenase 2 (MMP-8)	4QKZ	1.20 Å
Collagenase 3 (MMP-13)	2D1N	2.37 Å
Stromelysin 1 (MMP-3)	2D1O	2.02 Å
Stromelysin 2 (MMP-10)	1SLN	2.27 Å
Stromelysin 3 (MMP-11)	1HV5	2.60 Å
Matrilysin (MMP-7)	7WXX	1.50 Å
Metalloelastase (MMP-12)	2HU6	1.32 Å
Metalloproteinase (MMP-14)	5H0U	2.24 Å
Gelatinase A (MMP-2)	7XGJ	2.80 Å
Gelatinase B (MMP-9)	6EEM	2.61 Å

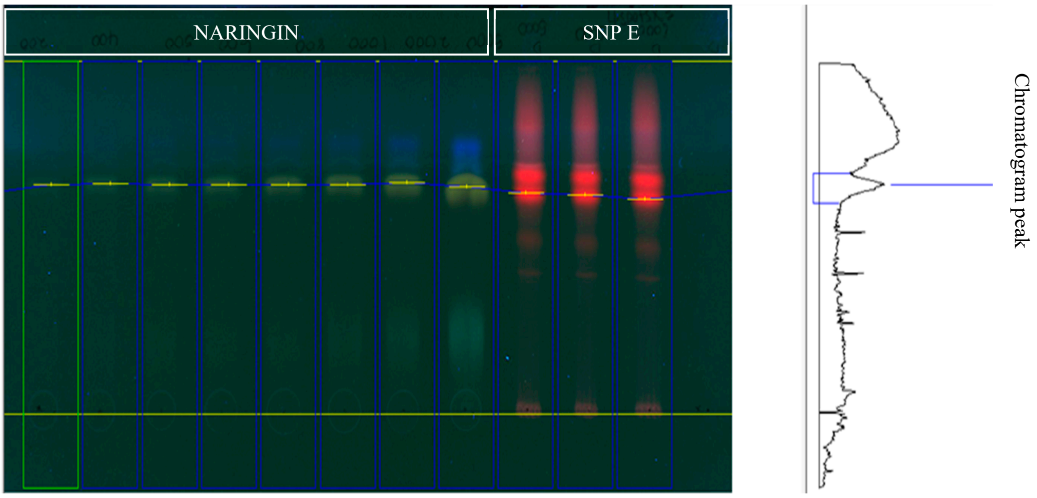

(a)

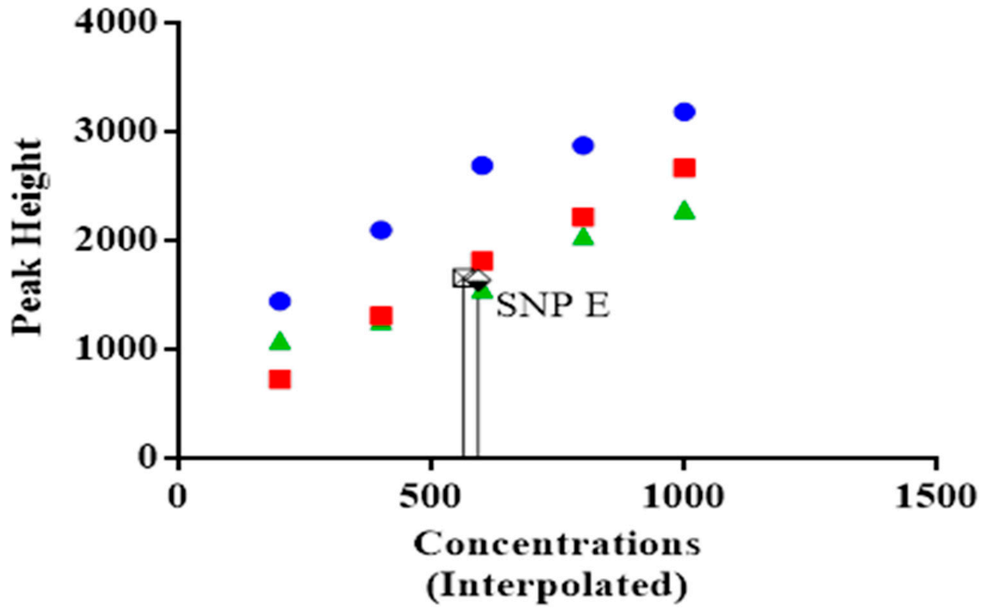

(b)

Figure S1. The HPTLC analysis of Naringin from SNP E of the plant extract. **(a)** Video scan capture of TLC plate at 366 nm of Naringin and SNP E and **(b)** Interpolation of the amount of Naringin in SNP E from the standard curve.

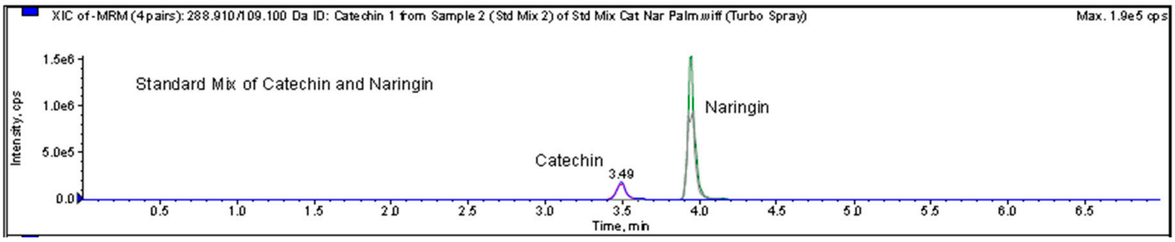

(a)

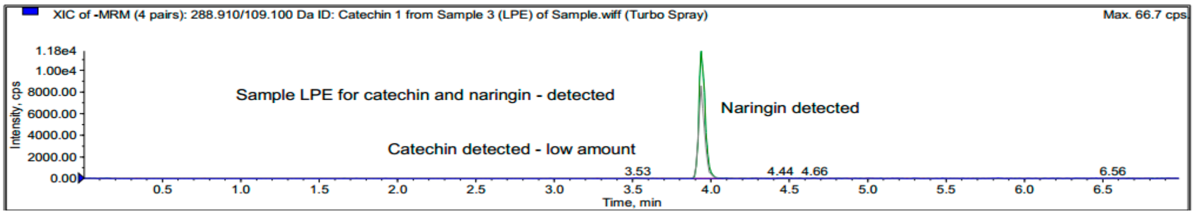

(b)

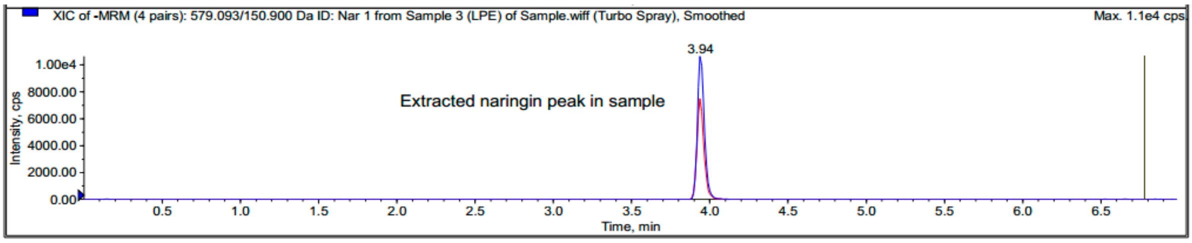

(c)

Figure S2. Targeted LC-MS/MRM analysis of SNP E against naringin and catechin. **(a)** Standard naringin and catechin peaks without the extract **(b)** Naringin and catechin peaks from SNP E and **(c)** Matching peaks of standard naringin and naringin from SNP E.

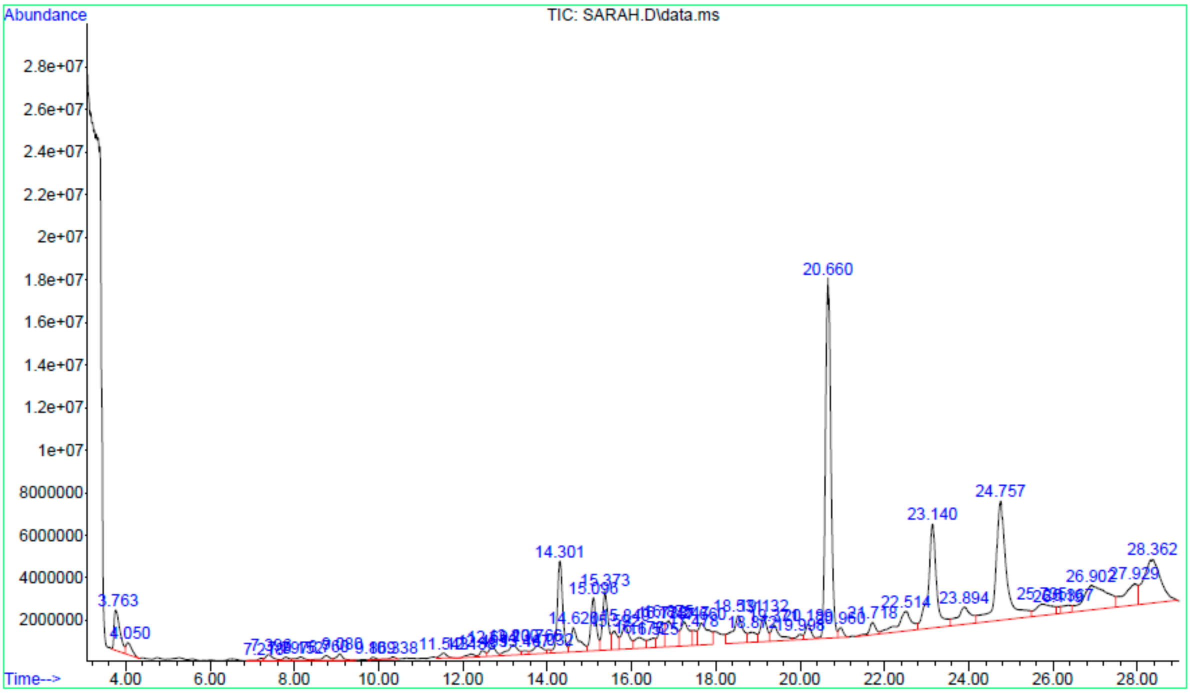

Figure S3. GC-MS analysis of SNP A from partially purified *M. pumilum* dichloromethane extract.
